# Manipulation of Innate and Adaptive Immunity by Staphylococcal Superantigens

**DOI:** 10.3390/pathogens7020053

**Published:** 2018-05-29

**Authors:** Stephen W. Tuffs, S. M. Mansour Haeryfar, John K. McCormick

**Affiliations:** 1 Department of Microbiology and Immunology, Schulich School of Medicine and Dentistry, Western University, London, ON N6A 5C1, Canada; mansour.haeryfar@schulich.uwo.ca; 2 Division of Clinical Immunology and Allergy, Department of Medicine, Western University, London, ON N6A 3K7, Canada; 3 Centre for Human Immunology, Western University, London, ON N6A 3K7, Canada; 4 Lawson Health Research Institute, London, ON N6C 2R5, Canada

**Keywords:** Superantigen, *Staphylococcus aureus*, T cell, Cytokine, Staphylococcal Enterotoxin, Toxic Shock Syndrome Toxin-1

## Abstract

Staphylococcal superantigens (SAgs) constitute a family of potent exotoxins secreted by *Staphylococcus aureus* and other select staphylococcal species. SAgs function to cross-link major histocompatibility complex (MHC) class II molecules with T cell receptors (TCRs) to stimulate the uncontrolled activation of T lymphocytes, potentially leading to severe human illnesses such as toxic shock syndrome. The ubiquity of SAgs in clinical *S. aureus* isolates suggests that they likely make an important contribution to the evolutionary fitness of *S. aureus*. Although the apparent redundancy of SAgs in *S. aureus* has not been explained, the high level of sequence diversity within this toxin family may allow for SAgs to recognize an assorted range of TCR and MHC class II molecules, as well as aid in the avoidance of humoral immunity. Herein, we outline the major diseases associated with the staphylococcal SAgs and how a dysregulated immune system may contribute to pathology. We then highlight recent research that considers the importance of SAgs in the pathogenesis of *S. aureus* infections*,* demonstrating that SAgs are more than simply an immunological diversion. We suggest that SAgs can act as targeted modulators that drive the immune response away from an effective response, and thus aid in *S. aureus* persistence.

## 1. Introduction

**Figure 1:**
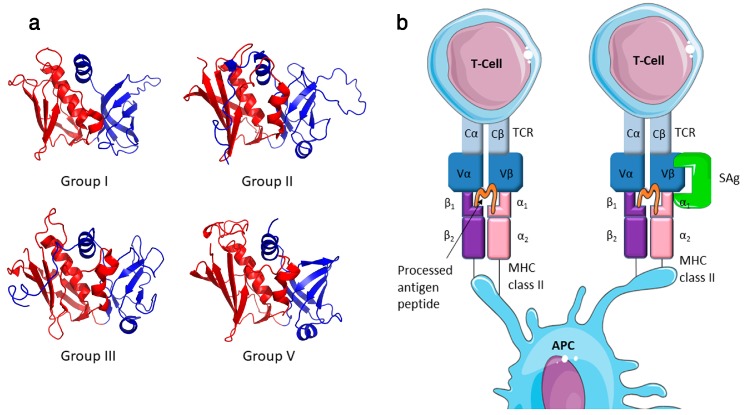
SAgs are two-domain proteins that activate T cell proliferation by binding in an unprocessed form to MHC class II and the TCR. (**a**) Ribbon cartoon showing the secondary structure of representative staphylococcal superantigens (SAgs). Examples represent each of the 4 major phylogenetic groups; Group I—TSST-1 (PDB: 4OHJ), Group II—SEB (PDB: 3SEB), Group III—SEA (PDB: 1SXT) and Group V—SElK (PDB: 2NTS). The colour defines the two-domain organisation of these proteins the N-terminal OB-fold shaded blue and the C-terminal β-grasp motif shaded red. (**b**) Conventional antigen presentation and specific T-cell activation results from antigen presenting cell (APC) presenting a processed antigen peptide on the MHC class II molecule which in turn is presented to a specific T-cell receptor (TCR). SAgs crosslink the MHC class II and TCR, unprocessed, and induce uncontrolled activation of T-cells. The SAg binds to the MHC class II outside the antigen presentation site and the variable beta (Vβ) chain of the T-cell receptor. The example interaction given here occurs between MHC class II α-chain and the low affinity site of the SAg (Cell illustrations are from Smart Servier medical art; https://smart.servier.com).

*Staphylococcus aureus* is an important bacterial pathogen of both humans and animals, and is responsible for a spectrum of conditions or diseases in its host species [1]. This multifaceted pathogen can produce an array of virulence determinants including surface-expressed matrix binding proteins (e.g. fibronectin-binding proteins), immune inhibitors (e.g. chemotaxis inhibitory protein of *S. aureus* (CHIPS)), various cytolytic toxins (e.g. α-toxin and leucocidins) and superantigens (SAgs) [2]. The staphylococcal SAg ‘family’ includes at least 26 genetically distinct paralogues (Table 1) encoded by *S. aureus* and other select staphylococcal species including *S. epidermidis*, *S. haemolyticus* and *S. pseudintermedius* [3,4,5,6,7,8]. These toxins are potent mitogens for T cells and induce dysregulated activation in a T cell receptor (TCR) Vβ-specific manner [3]. Staphylococcal SAgs range in size from 19 kDa to 29 kDa and have two major domains including an N-terminal domain, which displays a characteristic oligosaccharide/oligonucleotide binding (OB) fold, and a C-terminal domain that adopts a β-grasp motif (Figure 1). These two domains are divided by a structurally conserved α-helix, which spans the centre of the molecule [9,10].

Most characterized SAgs bind MHC class II and the TCR β-chain to form an unconventional T cell activation complex, which bypasses normal antigen processing and presentation to provoke a massive T cell response (Figure 1) [10]. T cell activation can be ‘forced’ by the SAg in a manner whereby peptide specificity of the T cell becomes irrelevant. There is also data to suggest that SAgs can also bind the costimulatory molecule CD28 and it co-ligand B7-2 contributing to the hyperactivity of the stimulated T cell [18,19]. Additionally, SAgs can activate T cells in a manner that is independent of the tyrosine kinase *Lck* [20], and recently this alternative T cell activation pathway has been linked to the α2 subunit of the extracellular matrix protein laminin (LAMA2), acting as a SAg co-receptor [21]. With the noted exception of staphylococcal enterotoxin H (SEH), which is a Vα-specific SAg [22], characterized SAgs each interact with the Vβ chain of the TCR resulting in stimulation of up to ~20% of the T cell population [3]. Although SAgs can engage Vβ chains using diverse orientations [23,24], recognition of the complementarity determining region (CDR) 2 loop appears to be the critical determinant for Vβ-specificity [25]. With respect to engagement of MHC class II, two distinct binding sites have been identified, and the presence of these sites can vary in different SAgs (Table 1). The first is referred to as the generic binding site, which is located within the SAg OB domain and which binds to the α-chain of MHC-class II. An additional MHC class II binding site can be found in the β-grasp domain of some SAgs, and interacts with the β-chain of the MHC class II molecule with zinc as a co-factor. This site is termed the high-affinity site, due to its ~100 fold greater affinity compared to the generic binding site [26,27,28]. 

The nomenclature of SAgs produced by *S. aureus* is based primarily on the emetic activity of these proteins [29]. Staphylococcal enterotoxins (SE) are the etiological agents of staphylococcal food poisoning in humans [30], which is characterised as an acute, self-limiting gastrointestinal illness with symptoms that last for around a day [30,31]. SAgs are generally heat and protease resistant and can also maintain their functional activity after exposure to weak acids and weak bleach solutions, thus allowing the SEs to survive the harsh conditions of the gut intact and cause emesis [30,32,33]. A SAg toxin is designated a bona fide SE if it can induce vomiting in a primate model (Table 1), and serotypes identified as SEs include SEA-E, SEG-I and SER-T [29,30]. The remaining SAgs have been designated SE-like (or SEl) as they are either weakly emetic or non-emetic, or still remain to be tested for such an activity (Table 1) [29]. The exception to this naming scheme is toxic shock syndrome toxin-1 (TSST-1), which was originally named both SEF and staphylococcal exotoxin C. TSST-1 was found to lack emetic activity and later renamed with reference to its association with toxic shock syndrome (TSS) [3]. *S. aureus* also encodes a group of 14 proteins that are structurally similar to SAgs and exhibit the classic SAg two-domain organisation but lack mitogenic activity for T cells [10]. These proteins were originally named staphylococcal exotoxin–like (SET) but have since been re-designated the staphylococcal superantigen-like proteins (SSls) to avoid confusion with the SEs and SEls [29]. Several of these 14 SSl proteins have been shown to possess a variety of immunomodulatory functions ranging from binding to complement and immunoglobulins (e.g. SSl7) [34] to interfering with neutrophil chemotaxis (e.g. SSl1 and SSl5) [35,36].

Despite a high level of structural conservation, staphylococcal SAgs vary a great deal in sequence identity. Based primarily on amino acid sequence divergence, these proteins can be organised into four phylogenetic groups (I–III and V, where group IV is composed entirely of related streptococcal SAgs). This grouping scheme reflects certain SAg characteristics such as MHC class II binding modes, and in some cases, disease associations [3,37]. Of note, group I SAgs (composed of SET, SElX, SElY and TSST-1), are very diverse and share limited homology with other staphylococcal SAgs and with each other [5,38]. Indeed, SElX shares as much sequence identity with the SSl proteins (27%) as it does with TSST-1 (24%) [38]. SET and the newly described SElY are more closely related to each other but have limited homology to other SAgs [5], thus potentially representing a separate linage of these toxins. To some extent, this group highlights the difficulties of classifying SAgs based on protein sequence identity and underlines the need to functionally characterise each protein.

Given the ubiquity of SAgs within clinical *S. aureus* isolates, this review will revisit the myriad of diseases associated with staphylococcal SAgs, but also consider recent evidence that may help to explain how these toxins play a role in *S. aureus* pathogenesis and to some extent colonisation. We will also consider the incredible diversity seen in the SAg family and discuss how this may contribute to the function of these toxins.

## 2. The Staphylococcal Superantigen Family

The distribution of SAgs in *S. aureus* is highly variable and each strain can encode between one and many of the 26 genetically distinct SAgs [3]. To date, two core genome-encoded SAgs have been identified and named SElW and SElX [38,39,40] whereas the majority of other SAgs are associated with mobile genetic elements (MGEs) such as plasmids, prophage and pathogenicity islands (Table 1) [11,17]. Indeed, the majority of the *S. aureus* pathogenicity islands (SaPIs) characterised to date encode at least one SAg gene [17]. A number of SAg genes have been associated with these elements (including *tst*, *seb, sec, sek*, *sell* and *seq*) and are found in different combinations amongst the *S. aureus* population [11,17]. Another example of an MGE-encoded SAg is SEA, which is present in the immune evasion cluster (IEC), an element made mobile by the β-toxin converting phage [41]. Outside of the core-genome encoded SAgs, the most prevalent paralogues reside within the enterotoxin gene cluster (*egc*), which is a group of SEls and SEs located within the VSaβ genomic island [42]. In total, seven different SAg genes have been discovered in this region including *seg, sei, selm, seln* and *selo*. The two remaining SAg genes have arisen from recombination; *selu*/*u2* are variants of an open reading frame formed from the recombination of two pseudogenes in the *egc* (*ψent1* and *ψent2*) and *selv* is the product of the recombination of *selm* and *sei* [14,43]. Four major variants of the *egc* have been identified with 5–6 functional genes present in each variant [11]. Approximately 80% of human nasal isolates were found to encode at least one SAg (in addition to SElW and SElX), and of this number, 50% were found to have the *egc*, suggesting that the majority of human clinical isolates encode at least 5 SAg genes [42].

To date, several virulence regulators have been shown to play a role in the regulation of SAg expression. TSST-1 has often been the focus of many of these studies, and it has been demonstrated that positive regulators of this toxin include the quorum-sensing accessory gene regulator (Agr) and the two-component sensing system *S. aureus* exotoxin expression (Sae) operon [44,45]. The transcription of *tst* also appears to be repressed (directly or indirectly) by a number of regulators that include SarA, the repressor of toxins (Rot) and carbon catabolite protein A (CcpA) (under high glucose conditions) [46,47,48]. The architecture of *seb* regulation appears to be similar to *tst*, in that it is positively regulated by *agr* and expression was antagonized by *rot* [49,50]. There is also data to suggest that Rot and SarA are not universal repressors of SAgs with both these regulators demonstrated to positively activate expression of *seh* [51]. The Sae locus has also been implicated in the expression of other SAgs in addition to *tst* including SEB and SElX [52,53,54]. Non-*egc* SAg genes tend to be transcribed at high optical densities *in vitr*o, however, the *egc* SAgs seem to only be expressed at lower optical densities [52,55]. This difference in growth phase expression suggests that these genes have different regulatory networks to other SAgs of *S. aureus,* although it has been suggested they are regulated in part by the transcriptional regulator alternative sigma factor (σ^B^) which may play a role in the expression of other SAgs such as *seh* and *tst* [52].

Given the large number of staphylococcal SAg genes, there are some discrepancies in the literature over the naming of the SE/SEl proteins. For example, it was suggested that SElU2, a highly similar variant of SElU, should be designated SElW as it arose through a separate recombination event from SElU [56]. However, SElU and SElU2 share a 94% sequence identity, have the same human Vβ activation profile (13.2 and 14) and are derived from the overlap of the same pair of pseudogenes (ψent1 and ψent2) making it unlikely that SElU and SElU2 would be encoded in the same strain [14,56,57]. With this understanding, it is our opinion that SElU2 is an allelic variant of SElU and does not merit a separate gene designation. Subsequently, through the analysis of multiple complete *S. aureus* genome sequences, Okumura and collegues identified a core-genome encoded putative SAg that shares closest identity to *sea* and *see* and also designated this gene *selw* [39]. To date, to our knowledge, no functional characterisation of *selw*, outside of its genome prevalence, has been performed. If this gene is expressed and the characterisitic properties of SAgs can be demonstrated, then to avoid confusion this gene should retain the designation *selw*.

With the known exception of SEH (which targets the TCR α-chain) [22], SAgs activate T cells by specifically interacting with variable region of the TCR β-chain [37]. As a result, it appears that each distinct SAg has a unique TCR Vβ-activation profile. Together, the staphylococcal SAgs characterised to date can activate the full human Vβ T cell repertoire, with the notable exceptions of Vβ4 and 11 (Table 1). Some SAgs, such as SEA, SEB and SEC, can activate 7 or more Vβ groups individually [15]. Thus, the diverse activation profiles of SAgs allow a combination of a few of these toxins to potentially stimulate a very large proportion of the total exposed T cell repertoire. In addition, a number of Vβ subfamilies are activated by more than one SAg, which has the potential to make some of these toxins redundant during infection if co-expressed (Table 1) [57]. Perhaps SAgs with overlapping activation profiles may be expressed at different times during infection allowing certain Vβ subtypes to be targeted throughout the infection process. Alternatively, TSST-1 is a highly potent SAg in humans although this toxin only activates the Vβ2 subfamily of T cells [15,58]. TSST-1 is widely believed to be the main, if not the sole, cause of the menstrual form of TSS [32] and thus the ability of a SAg to activate many TCR Vβ subfamilies does not necessarily correlate with the ability to cause serious disease. 

The reasons for the sequence diversity and potential functional redundancy of the staphylococcal SAg family have not yet been explained. One hypothesis is that to be able to bind highly variable molecules such as the TCR, SAgs too need to be diverse in order to reliably stimulate T cells. This may also be paralleled, in part, by the ability of SAgs to display binding preferences for different MHC class II molecules [59,60,61]. Another possibility is that the different toxin sequences allow for antigenic variation and exploit this to retain the ability to activate T lymphocytes even when neutralising antibodies are present to other SAgs. The existence of such a large group of antigenically distinct, highly potent, yet functionally conserved proteins suggests that the ability to stimulate and alter the course of adaptive immune responses is central to the evolutionary success of *S. aureus*.

## 3. Staphylococcal Superantigens and Disease

Overexpression of SAgs by *S. aureus* can trigger uncontrolled activation of T cells and the release of pro-inflammatory cytokines creating what has been termed a ‘cytokine storm’ [62]. This can lead to the development of TSS, which presents as a systemic disease with symptoms including sudden fever, hypotension and a diffuse macular rash, and potential progression to multiple organ dysfunction [63]. TSS is the major disease associated with SAgs, with menstruation-associated TSS (mTSS) as the best recognised variant [3,63]. Non-menstrual TSS can also occur as a result of the overproduction of SAgs during an invasive *S. aureus* infection [3]. mTSS has been primarily associated with improper tampon use. It has been postulated that certain tampons can unintentionally modify the vaginal environment, thereby stimulating the overexpression of TSST-1 from resident *S. aureus* [64]. TSST-1 production in the vaginal environment also appears to be heavily influenced by the vaginal microbiota, especially lactobacilli [65]. Species of lactobacilli have been shown to be able to inhibit the growth of vaginal *S. aureus* strains, and in addition to this, some lactobacilli can produce cyclic dipeptides that can reduce TSST-1 production from strains encoding this toxin [66,67]. This is important as studies of the vaginal microbiome indicate that microbial communities in the vagina can vary a great deal during menstruation [68]. The combined factors of conducive environmental conditions and changes in the microbiota, during menstruation, may allow resident *S. aureus* growth which in turn could lead to TSST-1 production. The TSST-1 SAg is understood to induce mTSS by translocating across the vaginal epithelium and stimulating local T cells [64,69,70]. The peak incidence of mTSS occurred in the early 1980s and was associated with a type of highly absorbent tampon. The subsequent withdrawal of this tampon from the market, as well as public education campaigns, warning labels on tampon products regarding mTSS, and proper use of tampons, has seen mTSS rates drop by up to 90% [71]. However, this disease is still reported and there has also been some recent cases of mTSS associated with the use of menstrual cups [72,73]. This underlines mTSS as a critical consideration in the design and application of feminine hygiene products. 

In addition to TSS, staphylococcal SAgs have been strongly associated with a number of other diseases including life-threatening conditions such as pneumonia and endocarditis [3]. SAgs in severe *S. aureus-*mediated pneumonia are likely to over activate the immune system, induce damage to the pulmonary epithelium, and subvert neutrophil activation resulting in serve inflammatory pathology [3,74,75]. Specifically, SEB, SEC, SElX and TSST-1 have all been shown to contribute to mortality in a rabbit model of necrotizing pneumonia [38,74,75]. Rabbit models of *S. aureus* infection have also demonstrated that SAgs can contribute to the disease process in the development of infective endocarditis, augmenting the formation and persistence of microbial heart valve vegetations [55,76]. It has been proposed that expression of SAgs in the blood can induce inflammation that leads to valve damage and capillary leakage, both of which can reduce blood flow and may contribute to vegetation formation [76]. 

Given that SAgs stimulate and manipulate the adaptive response in an uncontrolled manner, this may potentially prompt the immune system to target self-antigens [77]. In line with this hypothesis, many reports attribute staphylococcal SAgs as one of the potential causative agents in the development of a number of autoimmune conditions including psoriasis, atopic dermatitis, systemic lupus erythematosus, and potentially Kawasaki disease [78,79,80,81]. Recently, chronic exposure to SAgs has been implicated in the development of type II diabetes mellitus [82]. There is also data indicating that SEA can directly interact with adipocytes inhibiting the insulin response pathway, suggesting that this toxin could be a contributing factor in the onset of diabetes [83]. However, the etiology of these diseases is very complex, so caution is advised when assuming a role for SAgs in these conditions. The role that SAgs may play is still not clear but could range from initiating non-specific inflammation or enhancing a preexisting, pathological inflammatory process, to the forced activation of autoreactive T cells that had been controlled through peripheral tolerance mechanisms.

*S. aureus* is also responsible for an array of veterinary diseases, and SAgs have been associated with disease in non-human hosts, particularly cattle [84,85]. Indeed, carriage of SAg genes has been strongly associated with bovine intramammary infection (IMI) by *S. aureus* [86,87]. Bovine-specific variants of SEC, SElX and TSST-1 have all been demonstrated to induce Vβ-specific activation of bovine lymphocytes, suggesting that these toxins may perform similar roles in bovine and human hosts [38,88]. It has also been observed that higher concentrations of SEC is linked with more severe clinical bovine mastitis, which suggests that this toxin can contribute to the pathology of this disease [89]. Additionally, deletion of SElX attenuated infection in a model of *S. aureus*-induced bovine mastitis [90]. Together these studies demonstrate that staphylococcal SAgs are important contributors to the disease process during bovine mastitis and may be important for disease in other animal hosts that are sensitive to SAgs.

Diseases directly attributed to SAg intoxication (e.g., toxic shock syndrome) are thought to be a result of aberrant overexpression of these toxins leading to the severe pathology associated with uncontrolled activation of the adaptive immune response [62,63]. Nearly all clinical isolates of *S. aureus* encode two or more SAgs [40]. However, the incidence of severe SAg mediated disease is extremely low compared to the colonisation rates of SAg encoding *S. aureus* [16,40]. This indicates that the role of SAgs in colonisation and pathogenesis is subtler than simply generating a cytokine storm.

## 4. Superantigens in *S. aureus* Pathogenesis

**Figure 2:**
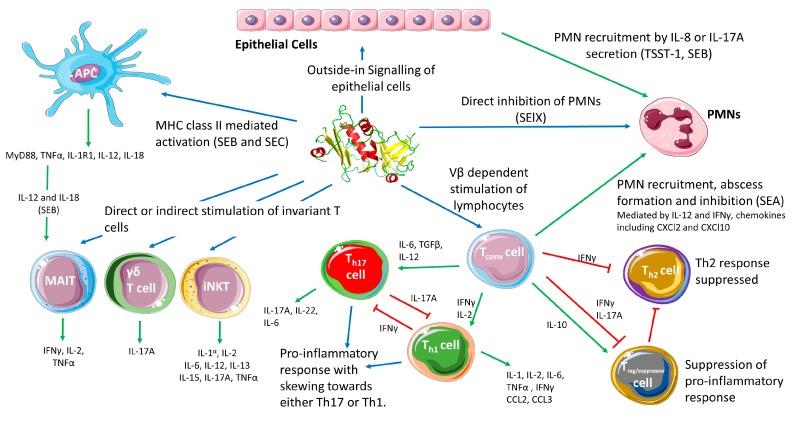
Schematic diagram showing the different cell types targeted by SAgs and the results of cellular activation. Blue arrows indicate direct mechanisms such as binding directly to cells resulting in activation. Green arrows indicate cytokine release or cytokine-mediated cellular activation. Red arrows indicate inhibitory or suppressive mechanisms (Cell illustrations are from Smart Servier medical art; https://smart.servier.com). IL: interleukin; TNF: tumor necrosis factor; IFN: interferon; MyD88: Myeloid differentiation primary response 88; CXCl: Chemokine (C-X-C motif) ligand; CCl: C-C motif chemokine ligand; PMN: polymorphonuclear leukocytes (granulocyte); MAIT; mucosa-associated invariant T cell; *i*NKT: invariant natural killer T cell.

The current paradigm for SAgs in *S. aureus* pathogenesis has these toxins playing a role of interference by stimulating inflammation, and creating an immunological ‘smoke screen’ which misdirects the immune system and potentially induces T cell anergy and deletion of certain Vβ subfamilies [91]. Conventional T cell (i.e., MHC-restricted CD4^+^ and CD8^+^ T cell) stimulation by SAgs results in the release of a variety of pro-inflammatory cytokines including interleukin (IL)-1, IL-2, IL-6, tumor necrosis factor (TNF)-α, interferon (IFN)-γ, and chemokines C-C motif chemokine ligand (CCL)2 and CCL3 (Figure 2) from a combination of T cells, antigen-presenting cells (APCs) and cells that are subsequently stimulated such as epithelial cells [3,77,92]. While a pro-inflammatory state of the immune system does seem to aid the bacteria, recent data indicates that immune system activation by SAgs may be more targeted than originally assumed. Inflammation is critical for the resolution of most bacterial infections. However, SAg driven inflammation appears to be able to subvert both activation and recruitment of important effector cells such as phagocytes promoting *S. aureus* survival. In this section we will briefly outline the role of T cells in protection against *S. aureus* infection, before considering how SAgs subvert the immune response, including targeted activities against conventional T cells, unconventional T cells and phagocytes. We will also discuss the potential role of SAgs in colonisation before finally reflecting on other *S. aureus* virulence determinants whose function may enhance or be enhanced by the activity of SAgs.

### 4.1. T cells in S. aureus Immunity 

It has been shown in mice that memory CD4^+^ T cells are protective against *S. aureus* during infection, eliciting a predominately Th1 response [93,94]. Brown and colleagues were able to demonstrate, in a murine model, that macrophages are also important in the resolution of *S. aureus* infection and antigen-specific CD4^+^ T cells were critical for macrophage recruitment. Of note is the fact that this study was also able to demonstrate that antigen-specific Th1 cells were activated in humans following bloodstream infection by *S. aureus*, indicating they may play a role in infection resolution [93]. *S. aureus* antigen-specific memory T cell responses are characterised by increased production of IFNγ and elevated expression of CCL5, which promotes the recruitment of monocytes and T cells to the site of infection [93]. T cells are also important in the recruitment of granulocytes such as neutrophils with both Th1 and Th17 activation pathways shown to be important for neutrophil recruitment and activity [94]. However, certain strains of laboratory mice are much more resistant to the effect of SAgs than both rabbits and humans [95]. This suggests that observations in conventional murine models used to assess T cell function, during experimental *S. aureus* infection, are likely made in the absence of subversion by SAgs. Nevertheless, T cells have been found to be critical in the recruitment of phagocytes needed to clear *S. aureus*. Therefore, it is not surprising that *S. aureus* has evolved a battery of toxins in response to manipulate the activity of T cells and secondary effector cell types. 

One of the major challenges associated with the study of SAgs is the availability of suitable models with which to investigate the role of these toxins during *S. aureus* infection. As mentioned, mice in general are more resistant to SAgs than humans, and physiologically irrelevant quantities of SAgs may be required to elicit a response [95]. Rabbits share a similar level of sensitivity to that of humans; however, mice are still favored as models due to lower costs and the availability of immunological tools to study the immune response [95]. The sensitivity to SAgs in mouse models has been addressed in part by the exploitation of transgenic animals expressing alleles of the class II human leukocyte antigen (HLA) [96]. Generally, SAgs have weak affinity for murine MHC class II molecules compared to human HLA molecules [97]. Using transgenic animals such as the C57BL/6 DR4tg that express the human DR4 HLA allele, SAg mediated disease such as TSS can be modelled as these animals become significantly more sensitive to direct exposure to purified SAgs such as SEB [98]. These animals can also be utilized in a bacteremia model for SAg expressing strains of *S. aureus*. When infected with strains such as Newman, at least 2 logs more bacteria can be recovered from the liver and hearts of C57BL/6 DR4tg mice compared to the SAg deficient strain [99]. One major caveat of these animals, however, is that different SAgs can display preferences for different types of HLA alleles [100]. Therefore, if a SAg of interest has low affinity for the HLA molecules expressed in a transgenic animal, then the model is likely to behave in the same way as an insensitive mouse. In addition, not all SAgs will be able to target mouse TCR Vβ chains, and in this case the model would also remain insensitive. Many of the studies that will be discussed in this review have utilized these partially ‘humanized’ HLA-transgenic murine models, due to the improved sensitivity to SAgs that these animals offer, and as a result have made important advances in our understanding of the role of SAgs in *S. aureus* pathogenesis.

### 4.2. Conventional T cell Responses to SAgs

The initiation of adaptive immunity requires antigen presentation to conventional CD4^+^ and CD8^+^ T lymphocytes, followed by many effector functions including the release of cytokines and chemokines [101]. Both Th17 and Th1 responses appear to be generally protective against *S. aureus*, and microbial clearance is primarily driven by Th1 effectors, with IFNγ production being critical for protection [93,94]. SAg-mediated T cell activation results in production of several cytokines, but IL-17A and IFNγ appear to be primary drivers of the early response to these toxins. In particular, the secretion of IL-17A has been traced to a subset of CD4^+^ effector memory T cells in the early stages of activation [102,103]. Blockade of IL-17A protects HLA-DR4 transgenic mice from SEB-induced TSS, preventing the secretion of downstream cytokines in the cascade including IL-6 and TNFα [103]. Thus, IL-17A overproduction appears to contribute to the pathology of TSS to drive the immune response in favour of a Th17 response, and away from a Th1 response, at least in the early stages of activation [103]. There is also strong evidence that SAgs can drive the activation of the Th1 response to a pathological level [104,105]. Together, these findings suggest that SAgs initiate a rapid overactivation of either inflammatory cascade and bias the immune system towards either a potentially pathological Th1 or Th17 response (Figure 2). This likely leads to an inappropriate recruitment and activation of effector cells, a detrimental impact on the clearance of *S. aureus* and consequently, contributes to bacterial persistence. This is supported by the observations made in an HLA-transgenic mouse model of bacteraemia. Deletion of *sea* in *S. aureus* strain Newman resulted in the reduction of bacterial burden and liver abscess formation. These data indicate that SAg induced neutrophil recruitment through the activation of T cells promotes S. *aureus* survival [99].

Activation of T cells by SAgs can also drive a suppressor or regulatory phenotype in both human CD4^+^ and CD8^+^ T cells, as well as directly stimulate the activity of FOXP3^+^ regulatory T cells (T_reg_), mediated primarily through the release of IL-10 [106,107,108,109]. In non-humanized mouse models of *S. aureus* infection, the induction of IL-10 appears to play opposing roles depending on the site of infection. This cytokine was protective during systemic infection, dampening potential pathological activation while conversely, during a local subcutaneous infection, IL-10 was found to support bacterial growth indicating that IL-10 production could benefit *S. aureus* [110]. Additionally, cell wall components of *S. aureus* can drive IL-10 production in a TLR2-dependent manner resulting in apoptosis of APCs and thus limiting SAg activity [111]. Furthermore, IL-10 expression can be driven by lower concentration of SAg than what is required to induce IFNγ production, indicating that SAgs can drive a suppressor or inflammatory phenotype depending in part on local SAg concentration (Figure 2) [108]. However, SAg induced T_reg_ cells express IL-10 at lower levels compared to those induced by IFNγ [107,108]. It is also noteworthy that the induction of T_reg_ cells is not protective against TSS, and FOXP3^+^ cells stimulated with SAgs also produce IFNγ and IL-17A once activated, mitigating their function as suppressors [106,112]. The overproduction of IL-17A and IFNγ induced by SAgs can also drive IL-10 induced suppressor T cells back to an effector state [112]. Together, this suggests that through coordinated expression of SAgs, *S. aureus* can manipulate the T cell response through differential activation of suppressor and effector T cells in a way that promotes infection by *S. aureus*. 

There have been a number of studies that indicate T cells becoming anergic following exposure to SAgs and that certain Vβ subsets can be reduced or deleted during infection [113,114]. The deletion or anergy of these subsets appears to target memory T cells, thus reducing the pool of antigen-experienced T cells [113,114]. Together, this would suggest that SAgs can overactivate T cells but eventually drive, at least partially, a form of immune suppression. However, these experiments have almost universally been performed in SAg-insensitive mouse strains (e.g. BALB/c) with large and potentially physiologically irrelevant quantities of purified toxin. Indeed, there is little evidence to indicate that conventional T cell anergy occurs at any level in the human system. In fact, there have been several observations from human studies that confound the theory that SAgs activate T cells to induce anergy and clonal deletion of T cells. Firstly, humans retain a diverse *S. aureus* antigen-specific pool of memory T cells [115]. This is unlikely to exist if memory T cells were constantly being driven into an anergic state by SAgs. Another key factor is the high prevalence of neutralising antibodies against staphylococcal SAgs, particularly the non-*egc* cluster SAgs such as TSST-1 and SElX [38,116,117,118]. This suggests that the helper T cell compartment is functional during and following *S. aureus* infection even when SAgs are expressed. Together this indicates that SAg-induced T cell anergy, observed in murine experiments, may be an artifact of exposure to large of quantities of SAg which are much higher than that encountered during more ‘realistic’ *S. aureus* exposure. 

The studies discussed here indicate that staphylococcal SAg activity may be far more ‘targeted’ than previously thought, and that by driving specific T cell activation pathways, these toxins can skew adaptive immune responses of the host away from a response that is generally protective against *S. aureus*. Additionally, the interference with cytokine responses created through exposure to staphylococcal SAg appears to have many important downstream affects for several cell types of the immune system, including phagocytes, which are critical in clearing *S. aureus* infections.

### 4.3. SAgs and Phagocytic Cells

Cytokines produced in the aftermath of SAg-induced activation of T lymphocytes can contribute to *S. aureus* pathogenesis by modulating the responses of other cells in the immune system. Neutrophils are recognized as critical innate immune cells for the clearance *S. aureus* [119]; however, it is well recognized that *S. aureus* has developed multiple strategies to avoid, subvert, limit or even kill these cells [119,120]. In the context of SAgs, SEA produced by *S. aureus* Newman induced the recruitment of neutrophils to the liver in a model of bacteremia in HLA-DR transgenic mice [99]. In this model, SEA-producing *S. aureus* induced higher levels of IL-12, IFNγ, TNFα and IL-6 in the liver, as well as the neutrophil chemokine C-X-C motif ligand 2 (CXCL2) (Figure 2). Although the mechanism is not entirely clear, bacterial survival decreased when *sea* was deleted indicating that enhanced abscess formation promoted by SEA may have been protective. Notably, CXCL2 has been shown to enhance intracellular survival of bacteria inside neutrophils [99,121]. Together this work demonstrated that SAgs can manipulate the recruitment and activation of phagocytes via the undesired stimulation of T cells, to promote the persistence of *S. aureus* during invasive infection. 

Neutrophils are not the only granulocytes affected following T cell activation by SAgs as granulocytic myeloid-derived suppressor cell (gMDSC) recruitment has also been noted [122]. Like neutrophils, these cells are recruited very early to the liver in an SEB-induced TSS model [99,122]. It is not yet entirely clear what role these cells play during TSS and *S. aureus* infection, although it is possible that the early recruitment of gMDSCs are protecting the liver from inflammatory damage [122]. MDSCs, whether granulocytic or monocytic, are thought to regulate the activity of T cells and reduce T cell proliferation [123]. It was noted, that in the TSS model, SEB-recruited hepatic gMDSCs were able to attenuate the proliferation of SEB stimulated *ex vivo* T cells, in a peroxide-dependent manner. These results were recapitulated in human cells and together suggest gMDSCs contribute to dampening down the proinflammatory response triggered by SAgs [122]. MDSCs are generated in response to a number of cytokines, including IL-1, IL-6, IL-10 and GM-CSF [124], all of which have been shown to be expressed as a consequence of SAg activation of T cells. It has also been suggested that MDSCs can regulate the activity of phagocytes such as macrophages and dendritic cells [124]. The activation and recruitment of these cells may thus contribute to the SAg-mediated survival of *S. aureus*, reducing the activity of recruited phagocytes [99]. By stimulating the recruitment of these cells, SAgs may induce suppression of phagocytes and T cells allowing *S. aureus* to benefit from anti-inflammatory mechanisms.

An alternative mechanism for neutrophil recruitment has also been suggested for TSST-1. It has been proposed that this toxin can directly interact with the vaginal epithelium and induce the release of IL-8 (Figure 2) [69]. This ‘outside-in’ signaling mechanism may explain the ability of TSST-1 to translocate across the vaginal epithelium [125]. By binding to epithelial cells and inducing IL-8 secretion, TSST-1 could induce the recruitment of neutrophils which migrate and disrupt epithelial membrane integrity allowing for the infiltration of the toxin and the induction of mTSS [69,125]. Outside the context of mTSS, SEB has been shown to induce inflammatory activation in nasal epithelial cells resulting in IL-17A expression, with particularly high levels of this cytokine being found in nasal polyps [126,127]. Acute sensitivity to SEB would perpetuate the persistent inflammation seen in nasal polyps, but also in normal epithelium, where IL-17A expression can activate pathways resulting in the recruitment of neutrophils [128]. It is possible that by generating proinflammatory cytokine release, SAgs can initiate barrier disruption through immune activation by targeting both epithelial and immune cells. This would allow *S. aureus* to breach host barriers through the disrupted epithelium generated by invading neutrophils.

The cross-linking of the TCR and MHC class II can activate both the T cell and cells expressing MHC class II, further promoting the release of a range of proinflammatory cytokines (Figure 2). This activation has been documented in different types of APC, including both monocytes and B cells. Engagement of MHC class II by SEB or SEC led to TNFα and IL-1β production through myeloid differentiation primary response 88 (MyD88) and NF-κB activation in these cells [129]. SEB is also capable of inducing the production of IL-12 from macrophages and thereby further augmenting T cell activation following MHC class II/TCR binding [130]. Osteoclast activation by SAgs has also been demonstrated, with TSST-1 triggering bone resorption by these cells directly [131]. While it is not clear if this phenotype is mediated through direct TSST-1-TCR/MHC class II binding, this is an interesting example of targeted pathology, mediated by SAgs, that has the potential to promote bone infection by *S. aureus* [131].

Some staphylococcal SAgs may be redundant during infection, resulting from overlapping Vβ activation profiles. Therefore, it is possible that these toxins have evolved alternative roles during infection. This has been demonstrated to be the case for SElX, which was shown to bind to and inhibit the function of neutrophils, and this activity was distinct from the ability of the toxin to induce T cell proliferation [54,75]. SElX is quite distinct from other members of the SAg family in that it does not form the classic two domain SAg structure [54]. However, SElX does contain a glycan-binding motif that allows engagement of glycoprotein receptors on the surface of neutrophils and monocytes, inhibiting the function of these cells (Figure 2) [54,75]. In a rabbit model of community-acquired MRSA necrotising pneumonia, it was this mechanism, and not the superantigenic potential of the protein, that contributed to pathogenesis, which further underscores the importance of *S. aureus* virulence strategies that target neutrophils [75]. It is interesting to note that this SAg has a Vβ activation profile that overlaps with 11 other SAgs, including SElK and SElQ, which are co-encoded with SElX in the USA300 lineage used in the rabbit pneumonia model [75,132]. This suggests that SElX is redundant as a SAg when co-expressed with others, and generally acts as a neutrophil inhibitor.

A picture has now emerged whereby phagocyte manipulation may represent one of the major roles of SAgs in *S. aureus* pathogenesis. Phagocytes, particularly neutrophils, appear to be a major terminal target of SAgs (Figure 2). By activating T cells, SAgs can stimulate cytokine responses that simultaneously recruit and then subvert the activity of these effector cells hindering the clearance of an *S. aureus* infection.

### 4.4. Unconventional T Cell Responses to SAgs

Considerable efforts have focused on understanding the stimulation of conventional (i.e., MHC-restricted CD4^+^ and CD8^+^) T cells; however, ‘unconventional’ T cells that harbor unique TCRs also represent important components of the human immune system. These include CD1d-restricted natural killer T (NKT) cells, mucosa-associated invariant T (MAIT) cells and γδ T cells, each of which appear to be activated directly or indirectly by SAgs (Figure 2) [98,133,134]. Unconventional T cells, in general, express a less variable TCR compared to conventional T cells, and have rapid effector responses once stimulated. Given the broad Vβ targets of the SAg family, it makes sense that some, but not all, SAgs could potentially target these T cell subsets. 

*i*NKT cells express a unique TCR that is composed of an ‘invariant’ α-chain, that preferentially pairs with a limited number of β-chains, primarily Vβ11 in humans, and Vβs 8.2, 7 and 2 in mice [135]. Consequently, select SAgs that target β-chains expressed by *i*NKT cells (e.g. SEB that targets mouse Vβ8), can directly activate *i*NKT cells and therefore can alter the host cytokine profile in an *i*NKT cell-dependent manner [134]. Indeed, HLA-DR4 transgenic mice depleted of *i*NKT were significantly less susceptible to challenge with SEB [98]. In this work, *i*NKT cells were important for production of an early wave of cytokines, including IL-17A and IFNγ, which triggered severe inflammation in mice with functioning *i*NKT cells [98]. Thus, specific staphylococcal SAgs can target and activate *i*NKT cells to promote a pathogenic response. 

MAIT cells are among the most abundant tissue-specific T cells and have been shown to be important in mucosal immunity and are potentially the first type of lymphocytes *S. aureus* encounters during infection [136]. Typically, MAIT cells are activated by the binding of the TCR to the MHC class I like molecule MR1 [137]. Recently, SEB has been shown to be able to stimulate MAIT cells by cross-linking the TCR on these cells and the MHC class II complex [133]. Activation of MAIT cells leads to high levels of IL-2, IFNγ and TNFα production. SAg activation also led to high level production of IL-12 and IL-18 from other cells, which in turn further stimulated the activation of MAIT cells (Figure 2) [133]. Interestingly, the secondary response to IL-12 and IL-18 appeared to be the dominant pathway in MAIT cell responses to SEB. Equally important, it was found that MAIT cells were no longer responsive to cognate bacterial antigens following SAgs exposure [133]. Therefore, SAgs can target and activate one of the most abundant peripheral T cell subsets, potentially subverting the activity of these innate-like T cells and diminishing their antimicrobial activity [133,138]. 

Gamma delta (γδ) T cells express a TCR composed of γ and δ chains that is distinct from the TCR found on αβ T cells. Activation of γδ cells, like *i*NKT and MAIT cells, does not require antigen presentation by MHC molecules [139,140]. Activated γδ T cells are both cytotoxic and capable of recruiting other immune cells through the production of cytokines including IL-17A [140]. These cells are found at low levels in peripheral blood and in lymphoid tissues but are found at much higher frequencies at mucosal barriers [140]. To date, it is still not entirely clear if γδ T cells are a direct target of staphylococcal SAgs, although a number of studies suggest that SAgs including SEA, SEB and TSST-1 can induce γδ T cell proliferation [141,142,143]. However, it is hard to rule out indirect stimulation by SAgs via αβ T cells as peripheral blood mononuclear cells (PBMC) are present in experiments showing proliferation of these cells [141,142]. Even when direct stimulation can be demonstrated, it appears that γδ T cells are orders of magnitude less sensitive to SAgs compared to αβ T cells [143]. More recent animal studies have shown that in the host, SAg treatment results in the activation of γδ T cells. Whether activation is direct is not entirely clear, but prominent responses can be detected in models utilising SAg delivery to mucosal surfaces [144,145]. Rats challenged orally with SEB demonstrated a detectable increase in CD3^+^ cells including, specifically, γδ cells [144]. Further to this, γδ T cells, like *i*NKT cells, appear to be an early source of IL-17A. In mice receiving a respiratory challenge of SEA, activation of αβ T cells led to subsequent activation of γδ T cells, which in turn contributed to the production of IL-17A and led to rapid neutrophil recruitment [145]. These two animal studies collectively indicate that despite any clear direct interaction between SAgs and γδ T cells, SAg-mediated immune activation can still subvert the proper activation of these cells and contribute to immune cell perturbation at the mucosal barrier. 

### 4.5. SAgs in Colonisation

There are multiple lines of evidence to suggest that SAgs are important for *S. aureus* colonisation of the human host. First, antibodies that recognize SAgs can be found in persistent carriers of *S. aureus*, suggesting that these toxins are expressed in vivo during colonisation [146,147]. Furthermore, SAg transcripts have been detected in nasal swabs of human carriers [146]. Using a nasal colonisation model in HLA-DR4 transgenic mice, deletion of *sea* (in strain Newman) and deletion of *seb* (in strain COL) resulted in an increased *S. aureus* burden within the nasal cavity [148]. This work suggested that SAgs may act as ‘checkpoints’ to promote inflammation in order to prevent bacteria from reaching pathogenic densities, which could explain why numbers of *S. aureus* cells are relatively low during human nasal colonisation [148,149]. As discussed earlier, SEB can induce the production of IL-17A from human nasal epithelium, which could initiate the process, as postulated here, in humans [127,128]. In addition, work in non-humanized mice found that resolution of *S. aureus* colonization in mice is T cell-mediated, with Th17 cells dominating the response and with a heavy influx of neutrophils to the nasal cavity [150]. As this experiment was performed in non-humanized mice, it is again unlikely that SAgs are contributing to this immune activity; however, along with observations from humans, there appears to be a clear link between Th17 responses and the control of *S. aureus* numbers during colonisation. 

Other potential mechanisms underlying colonisation could involve direct manipulation of the immune system by SAgs to promote persistent carriage. It has been shown in humans that a high Th1 to Th17 response ratio is linked with non-carriage of *S. aureus* and the reverse is seen in persistent carriage [151]. This suggests the Th17 response is less important in clearing colonising *S. aureus*. Although a Th1 response was linked with non-carriers, a bias towards Th17 in a persistent carrier may represent an attempt by the host to clear the established colony and therefore is consistent with animal studies. As previously discussed, the Th1-mediated response is protective against *S. aureus* during infection, and this also appears to be the case in colonisation, with Th1 regulated human β-defensin 3 being suggested as one of the major effectors [93,151]. It should be noted that it remains unclear if this bias is necessarily driven in any capacity by the colonising *S. aureus*. If a bacterium-directed mechanism is involved, SAgs represent the most likely candidate given their ability to directly alter the host’s adaptive response. 

Collectively, the above studies suggest that *S. aureus* colonisation promotes a Th17-mediated response in the nasal cavity. SAgs may be important drivers of response in order to maintain low bacterial numbers, prevent overt host pathology, and maintain an overall environment that is suitable for continued asymptomatic carriage. Finally, the immune evasion strategies previously discussed could contribute to the adaptive immune evasion and prevent complete clearance of colonising *S. aureus* resulting in persistent carriage.

### 4.6. SAgs’ Cooperation with Other S. aureus Virulence Determinants

SAgs are only one of the many different types of secreted virulence factors produced by *S. aureus*. Other *S. aureus* exoproteins include lytic toxins, degradative enzymes, and inhibitors of both cellular and humoral immune responses. During infection, these factors will be co-expressed and contribute collectively to the infective processes. By evaluating distinct activities within multifunctional proteins, we can characterize potential relationships between different virulence determinants and examine how SAg function can support, or be supported by, the activity of other factors from *S. aureus*.

As previously stated, SEA can contribute to the subversion of neutrophil responses in the host via the activation of T cells. It is also interesting to note that SEA is often found co-encoded with the neutrophil inhibitor CHIPS on the IEC, an important locus for *S. aureus* infection in humans [41]. This could suggest that co-expression of SEA and CHIPS can coordinate a T cell-dependent inflammatory signature that subverts engagement of the bacteria by neutrophils, impeding the ability of the immune system to clear the infection.

The high prevalence of neutralising antibodies against staphylococcal SAgs in the human population suggests that many of these proteins are quickly inactivated during infection. However, *S. aureus* can express staphylococcal protein A (Spa) and the second immunoglobulin-binding protein (Sbi), which both bind to the Fc region of IgG and interfere with the functional properties of these immunoglobulins, including disrupting the opsonisation of *S. aureus* bacterial cells [152,153]. During growth, Spa and Sbi are released from the cell wall of *S. aureus*; these secreted forms could bind IgG targeted against secreted factors including SAgs [152,154]. Other *S. aureus*-secreted factors that bind immunoglobulins include staphylococcal superantigen-like proteins 7 and 10 (SSl7 and SSl10). SSl7 has been shown to be able to bind IgA, and like Spa and Sbi, SSl10 can bind the Fc region of IgG1 [155,156]. Together, these multiple factors may contribute to a reduction in the function of neutralising antibodies in the immediate vicinity of the invading bacteria. This would allow SAgs to target T cells entering the infected area without interference from circulating antibodies, while concurrently the systemic presence of neutralising antibodies could protect the host from wide-spread immune activation mediated by the SAgs. 

SAgs, together with the leucocidins, could contribute to mucosal epithelial barrier disruption. For example, direct damage and inflammation to the vaginal epithelium can be induced by α-toxin (Hla) and γ-toxin (both HlgAB and HlgCB), potentially contributing to the mTSS disease processes [157,158]. In the case of Hla, expression of this toxin was shown to augment the translocation of TSST-1 in a porcine *ex vivo* vaginal epithelial model, which would allow TSST-1 to induce further inflammation once across the epithelium [158]. Here, the outside-in signaling mechanism may also contribute to epithelium disruption by stimulating IL-8 resulting in the chemoattraction of neutrophils [125]. The result of this perturbed mucosal barrier could allow *S. aureus* cells to invade across the epithelial barrier. Additionally, sublytic concentrations of cytolytic toxins including Hla, PVL and LukAB have all been found to activate the intracellular NOD-like Receptor (NLR) protein 3 (NLRP3) inflammasome in neutrophils, monocytes and macrophages, leading to activation of Caspase 1-dependent pro-inflammatory cytokines such as IL-1β and IL-18, and induction of necrotic cell death [159,160,161]. Inflammation induced by these toxins could further contribute to SAg activity via the recruitment of lymphocytes that can be in turn targeted by secreted SAgs. This suggests that together, SAgs and cytolytic toxins elicit pro-inflammatory pathology that induces the recruitment of the other protein’s target cells and can cooperatively amplify the toxic effect to the benefit of the invading bacteria. 

## 5. Concluding Remarks

The staphylococcal SAg family is a diverse group of immunomodulatory toxins that can stimulate the majority of conventional, and sometimes unconventional, human T cells in our repertoire through targeting of the Vβ region of the TCR. These toxins are ubiquitous in human clinical isolates of *S. aureus*, implying that they are critical for *S. aureus* pathogenesis and colonization. Moreover, the group exhibits extensive sequence diversity, which likely contributes to the evasion of the humoral immune system, and improved molecular targeting of the TCR and MHC class II. While it has been proposed that staphylococcal SAgs are acting as an immunological diversion through the induction of a pro-inflammatory immune response, recent research has demonstrated that these toxins are likely more targeted than previously appreciated. By varying concentration and temporal expression, SAgs potentially afford *S. aureus* the ability to manipulate the adaptive immune response in a direction that suits its niche. There is a growing body of evidence that the innate immune response is a downstream target of SAg activity with critical effector cells such as neutrophils and macrophages subverted by the activity of these toxins. Altogether, the SAgs are have evolved in *S. aureus* to target T cells and their effectors, subverting their activity to alter the course of the immune response in a way that benefits *S. aureus* persistence. 

## Figures and Tables

**Table 1:** Properties of the staphylococcal superantigen family.

SAg	Phylogenetic Group	Molecular Mass (kDa)	Emetic	Human Vβ Specificity	MHC Class II Binding ^a^	Associated Mobile Genetic Elements
SEA	III	27.1	+	1, 5, 6, 7, 9, 15, 16, 18, 21, 22, 24	α + β	ɸSa3n
SEB	II	28.3	+	1, 3, 6, 12, 13.2, 14, 15, 17, 20	α	SaPI
SEC	II	27.5	+	3, 12, 13.2, 14, 15, 17, 20	α	SaPI
SED	III	26.4	+	1, 3, 5, 8, 9, 12, 14	α + β	Plasmid (pIB485-like)
SEE	III	26.4	+	5, 6, 8, 9, 13.1, 16, 18, 21	α + β	Integrated Plasmid
SEG	II	27	+	3, 12, 13, 14, 15	α	vSAβ (*egc*)
SEH	III	25.2	+	Vα8, Vα10	β	ɸSa3mu
SEI	V	24.9	+ ^b^	1, 5, 6, 23	β	vSAβ (*egc*)
SElJ	III	28.5	NK	8, 21	α + β	SaPI/ɸSa3n/Plasmid (pF5/pIB485-like)
SElK	V	26	-	1, 5, 6	β	SaPI
SElL	V	26	-	1, 5, 7, 16, 22, 23	α + β	SaPI
SElM	V	24.8	+ ^b^	8, 9, 18, 21	α + β	vSAβ (*egc*)
SElN	III	26.1	+ ^b^	7, 8, 9, 17	α + β	vSAβ (*egc*)
SElO	III	26.7	+ ^b^	5, 7	α + β	vSAβ (*egc*)
SElP	III	27	+ ^b^	5, 8, 16, 18, 21	α + β	ɸSa3n
SElQ	V	28	-	6, 21	α + β	SaPI/ɸSa3n
SER	II	27	+	3, 12, 14	α	Plasmid (pF5/pIB485-like)
SES	III	26.2	+	9, 16	α + β	Plasmid (pF5)
SET	I	22.6	+ ^b^	NK	α	Plasmid (pF5)
SElU/U2	II	27.1	NK	13.2, 14	α	vSAβ (*egc*)
SElV	V	25	NK	6, 18, 21	α + β	vSAβ (*egc*)
SElW	III	27.3	NK	NK	NK	Chromosomal (Core genome)
SElX	I	19.3	NK	1, 6, 18, 21	NK	Chromosomal (Core genome)
SElY	I	22.5	+^c^	NK	NK	Chromosomal
SElZ	II	27.0	NK	NK	NK	Chromosomal
TSST-1	I	22	-	2	α	SaPI

Data compiled from references: [3,4,5,10,11,12,13,14,15,16,17], (NK) Not known, ^a^ (α) low affinity site α-chain or (β) high affinity site β-chain, ^b^ Weakly emetic i.e. can only induce infrequent emesis at dose ≥ 100µg/kg in primates [11,12], c. Emesis demonstrated only in *Suncus murinus* rodent model and not primates. SAg: superantigen; MHC: major histocompatibility complex; TSST-1: toxic shock syndrome toxin-1; SE: staphylococcal enterotoxin; SEl: SE-like protein.
